# Cardiac implantable electronic device infections: Observational data from a tertiary care center in Lebanon

**DOI:** 10.1097/MD.0000000000014906

**Published:** 2019-04-19

**Authors:** Marwan Refaat, Patrick Zakka, Maurice Khoury, Hassan Chami, Shareef Mansour, Bernard Harbieh, Bernard Abi-Saleh, Abdul Rahman Bizri

**Affiliations:** a Department of Internal Medicine, Division of Cardiology, American University of Beirut Medical Center, Beirut, Lebanon; b Department of Internal Medicine, Emory University Hospital, Atlanta, GA, USA; c Department of Internal Medicine, Division of Cardiology, Keserwan Medical Center; d Department of Internal Medicine, Division of Infectious Diseases, American University of Beirut Medical Center, Lebanon.

**Keywords:** cardiac implantable electronic device, cardiac implantable electronic device infection, implantable cardioverter defibrillator, pacemaker

## Abstract

With increasing rates of device implantation, there is an increased recognition of device infection. We conducted a retrospective observational study in a tertiary care center in Lebanon, with data collected from medical records of patients presenting with cardiac implantable electronic device (CIED) infection from 2000 to 2017 with the purpose of identifying etiologies, risk factors and other parameters, and comparing them to available data from the rest of the world. We identified a total of 22 CIED infections. The most common microbial etiologies, including involvement in polymicrobial infection, were coagulase-negative staphylococci (45.5%) and *Staphylococcus aureus* (22.7%). Rare cases of *Brucella melitensis*, *Sphingomonas paucimobilis*, and *Kytococcus schroeteri* device infection were seen. Heart failure was seen in 77.3% of patients, hypertension in 68.2%, and chronic kidney disease in 50%. Skin changes were the most common presenting symptoms (86.4%). Antibiotics were given to all patients and all had their devices removed, with 36.4% undergoing new device implantation. This is the first study of CIED infections in Lebanon and the Middle East. Local epidemiology and occupational exposure must be considered while contemplating the microbial etiology of infection. Close monitoring after device implantation is important in preventing device infection that carries high risk of morbidity and mortality.

## 1 Introduction

The use of cardiac implantable electronic devices (CIEDs) in cardiovascular disease management has rapidly increased over the past 50 years, largely due to improved device technology^[1]^ and growing indications for CIED implantation. The overall number of CIED implantations in the United States increased by 96% from 1993 to 2008.^[2]^ CIEDs are used to regulate cardiac rate and rhythm and coordinate myocardial contractions. Overall, they have been shown to improve symptoms, quality of life and survival in appropriately selected patients.^[3]^ The reported incidence of CIED infection varies significantly between different studies. A review of over 20 studies showed that the rate of CIED infection ranges from 0.68% to 5.7%,^[4,5]^ and up to 19.9% in studies conducted in the 1970s/1980s.^[6]^ CIED infections can be categorized into pocket infections and deeper infections. Pocket infection involves the subcutaneous pocket harboring the CIED along with the subcutaneous lead segments.^[4]^ Deeper infection involves the transvenous lead segment, and is usually associated with bacteremia and possibly endovascular infection. It includes vegetations that may occur on the intracardiac segments of the leads or device-related endocarditis (vegetations also present on the neighboring endothelium or tricuspid valve).^[4]^ Multiple risk factors have been implicated in the development of CIED infection. Diabetes mellitus, heart failure, and renal failure have been shown to be strongly related to CIED infection.^[7]^ Other host-related risk factors include age (>60 years), chronic obstructive pulmonary disease, use of oral anticoagulation,^[8]^ immunosuppression, and long-term corticosteroid usage.^[9]^ Procedure-related risk factors include: presence of more than two pacing leads,^[9]^ fever within 24 hours of device implantation, prolonged hospitalization, peri-procedural temporary pacing, and early re-intervention.^[10]^ The type of device used may affect the rate of infection. Several studies have demonstrated that more complex devices are at higher risk of infection.^[11]^ One study showed that there is higher risk of infection in dual chamber systems versus single-chamber systems, but another study showed no difference in rate of infection between the two.^[12]^ Operator characteristics and operator number are also important risk factors. One study demonstrated higher incidence of implantable cardioverter defibrillator (ICD) infection with devices implanted by physicians with history of low implantation volume.^[12]^ Rates of infection and complications were also shown to be higher when a single operator performed the procedure compared to two operators.^[13]^ CIED infection is considered a major challenge to the entire medical system, especially with the increasing use of such devices. The impact that device infection has on morbidity and mortality remains significant despite the growing experience in how to manage such cases. Part of the solution is to be made aware of CIED infection etiologies, risk factors, and patient presentations which would ultimately translate to more prompt and efficient patient management. There is limited data on CIED infections in Lebanon and the Middle Eastern region and this is to our knowledge the first study that presents data on device infections from a tertiary care center in the region. We additionally aim to compare this data to data from other centers.

## 2 Methods

The setting in which data was collected is the American University of Beirut Medical Center, a tertiary care medical center in Beirut, Lebanon. The design of this study is a retrospective, single-center observational study, with data collected from medical records of patients presenting with CIED infection from the years 2000 to 2017. Subjects were screened for parameters that fit the definition of CIED infection, including: pocket infection, endovascular infection, device-related endocarditis, and bacteremia. Patients were electronically identified using the International Classification of Disease, Ninth Revision, codes for “infection, inflammatory reaction, and other complications due to cardiac implant and graft.” Patients would be considered eligible if they satisfied the strict definition of CIED infections.

After obtaining the approval from the Institutional Review Board, hospital medical charts of all patients were individually reviewed for demographic data (date of birth, gender, age at diagnosis), clinical data (smoking status, associated co-morbidities, presenting symptoms), laboratory and microbiological data (blood cultures, device site cultures, complete blood counts, and inflammatory markers), and treatment options undertaken (medical and/or surgical). Other parameters included are the type of device implanted, time of implantation and pre-procedural antibiotic use. This simple retrospective method of collecting data from already established patients who have had CIED infections through disease coding leaves little room for potential bias.

## 3 Results

A total of 22 CIED infections were identified in patients presenting between the years 2000 to 2017 and all 22 patients were included in this study. No patients had any missing data and follow-up was not an issue. Simple percentages of descriptive observational data are presented. Thirteen (59.1%) patients had their devices implanted at the study's tertiary care center while the remaining 40.9% of patients had their devices implanted at outside hospitals. An estimate of CIED infection rate could not be calculated as many patients who had their devices implanted at the study's center were lost to follow-up. Nineteen cases (86.4%) were seen in males (mean age 62.9 SD 20.1) while three cases (15%) were seen in females (mean age 65.7 SD 13.5). Ages of the patients studied ranged from 9 months to 85 years. Sixteen patients (72.7%) were above the age of 60 upon presentation. In considering duration after implantation after which infection occurred, 9.1% suffered from infection 0 to 28 days after implantation, 13.6% between 28 and 356 days after implantation, and 77.3% after more than 365 days of implantation.

Twelve patients (54.5%) had CIEDs implanted for history of ischemic cardiomyopathy, four (18.2%) for complete heart block, two (9.1%) for nonischemic cardiomyopathy, one (4.5%) for Mobitz type II atrioventricular block, one (4.5%) for Brugada Syndrome, one (4.5%) for symptomatic bradycardia, and one (4.5%) for an unknown arrhythmia. The types of devices implanted included ICDs, cardiac resynchronization therapy pacemakers (CRTPs), cardiac resynchronization therapy defibrillators (CRTDs), dual-chamber pacemakers and single-chamber pacemakers. The most common device infected was the ICD, seen in nine patients (40.9%). CRTDs were infected in six patients (27.3%), dual-chamber pacemakers in five patients (22.7%), a CRTP in one patient (4.5%), and a single-chamber pacemaker in one patient (4.5%). All patients were given antibiotic prophylaxis prior to their device implantation.

**Figure 1:**
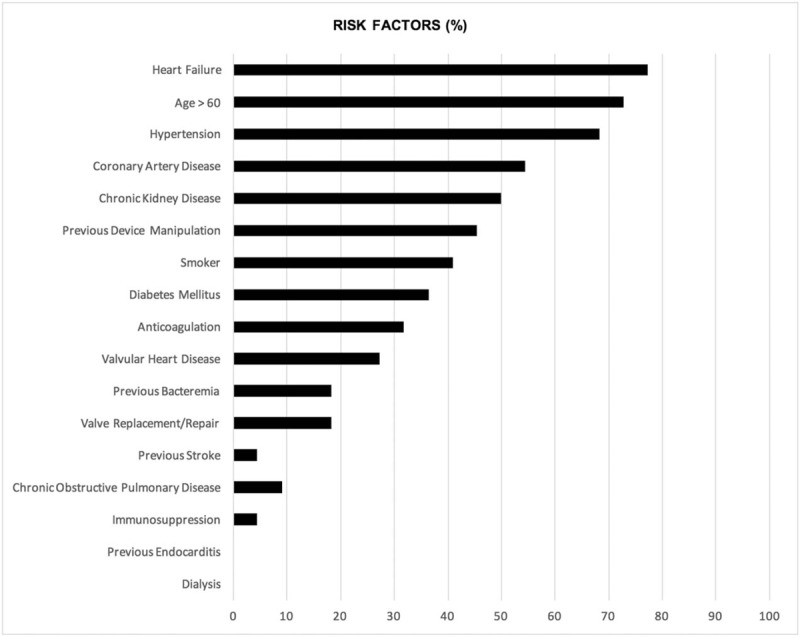
Risk factors and comorbid conditions present in patients presenting with cardiac implantable device infection in tertiary care center in Lebanon from 2000 to 2017.

The most common risk factors or comorbidities seen in this patient sample were heart failure (77.3%), age above 60 (72.7%), hypertension (68.2%), coronary artery disease (54.5%), chronic kidney disease (CKD) (50%), previous device manipulation (45.5%), current smoking (40.9%), and diabetes mellitus (36.4%). Previous device manipulation included device upgrades and lead changes. Only one patient was immunosuppressed due to chronic lymphocytic leukemia (CLL). The remaining risk factors are shown in Figure 1.

Nineteen patients (86.4%) presented to the hospital for skin changes at the CIED site and five (22.7%) patients had tenderness in that region. Fever was a presenting symptom in five patients (22.7%) and two (9.1%) patients presented in shock. Complete blood counts were taken for all patients as part of the work-up for infection. White blood cell counts ranged from 4500 to 22,300 cells/μL, with five (22.7%) patients presenting with elevated levels of white blood cells. Inflammatory markers considered included erythrocyte sedimentation rate (ESR) and C-reactive protein (CRP). ESR was increased in five (22.7%) patients and CRP in seven (31.8%). Overall, ten (45.5%) patients had elevation in at least one of these inflammatory markers. Blood cultures were taken for all patients and eight (36.4%) were positive for bacteremia.

**Figure 2:**
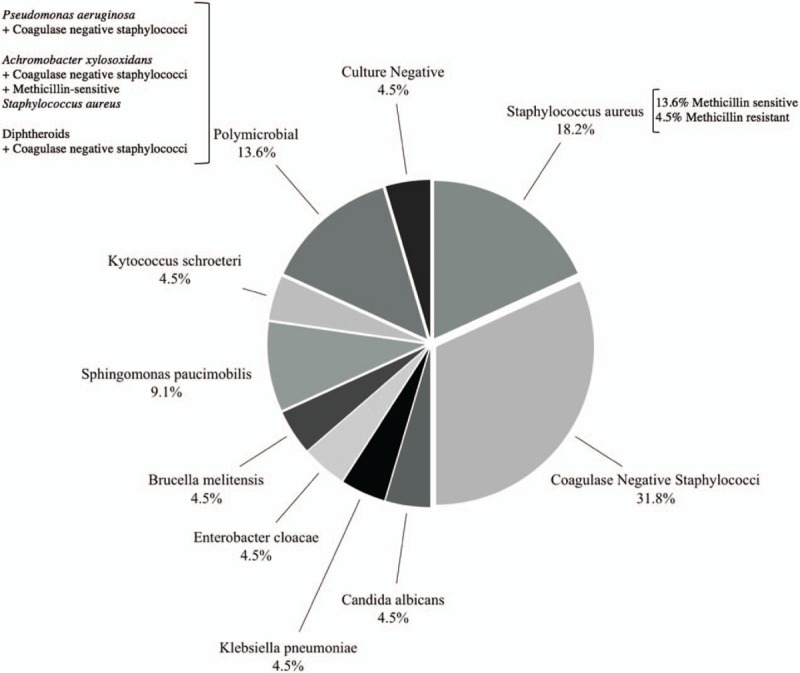
Cardiac implantable electronic device infection etiologies in 22 recruited patients in tertiary care center in Lebanon.

Thirteen (59.1%) patients were diagnosed with pocket infections while the remaining nine (40.9%) had deeper infections, three of which had associated infective endocarditis. Cultures from the sites of infection were taken from all patients: one (4.5%) case was culture-negative, four (18.2%) due to *S aureus* (three methicillin-sensitive *S aureus* [MSSA] and one methicillin-resistant *S aureus* [MRSA]), seven (31.8%) due to coagulase-negative *Staphylococcus* (CoNS), one (4.5%) due to *Candida albicans*, one (4.5%) due to *Enterobacter cloacae*, one (4.5%) due to *Klebsiella pneumoniae*, one (4.5%) due to *Brucella melitensis*, two (9.1%) due to *Sphingomonas paucimobilis*, one (4.5%) due to *Kytococcus schroeteri*, and three (13.6%) due to polymicrobial infection. Taking polymicrobial infections into account, CoNS was actually isolated from 45.5% of cultures and *S aureus* from 22.7%. A summary of the etiologies is shown in Figure 2, including the components of the polymicrobial infections.

As mentioned earlier, on presentation, eight patients (36.4%) had bacteremia. Of the eight patients, five had bacteremia with the same organism as that cultured around their devices. These included two of the CoNS cases, one *E cloacae* case, one *S paucimobilis* case and one *K schroeteri* case. The remaining three cases with positive blood cultures were Diphtheroids in a CoNS case, CoNS in the *K pneumoniae* case, and MSSA in blood despite negative cultures from the device. Additionally, four (18.2%) patients in the total sample had prior history of bacteremia, but only one of them presented with infection of the same organism (a CoNS case).

In all patients, antibiotics were used in the management of CIED infection. Vancomycin was administered in 77.2% of the cases. Doxycycline and rifampin were given to the patient with *B melitensis* CIED infection and ceftazidime and ciprofloxacin were used for the treatment of *S paucimobilis* CIED infection. All 22 patients had their devices removed after infection and eight (36.4%) of them had their devices replaced. One patient with *S paucimobilis* infection underwent extraction of ICD and replacement with a subcutaneous ICD. Of the 22 patients, one patient, who presented with shock and *E cloacae* device infection, passed away after device extraction. All other cases were successfully managed with antibiotics (and antifungal in the *C albicans* case) and were followed up as outpatients.

## 4 Discussion

In this retrospective single-center observational study at a tertiary care center in Lebanon, a total of 22 patients that presented for CIED infection from the years 2000 till 2017 were identified. A variety of causative agents of CIED infection were noted. The most commonly encountered organisms in this study, taking into account polymicrobial infections, were *S aureus* (22.7%) and coagulase-negative staphylococci (45.5%). This parallels available data on etiologies of CIED infection in the literature. CIED infections can be caused by virtually any pathogen. However, in most reported series, 60% to 80% of infections were caused by staphylococcal species, with CoNS being the most common pathogen in some studies and *S aureus* in others.^[1,3,4,6]^ CoNS and *S aureus* are common pathogens because they are part of the skin flora. Because of this, it is necessary to take repeated cultures and aspirates to differentiate between the patient's skin flora and pathogenic organisms.^[14]^ Polymicrobial infection is also commonly seen and generally involves one or more species of CoNS, as in all three of the polymicrobial cases in this sample. We reported one case of methicillin-resistant *S aureus*, and it has been seen in 4% to 22% of studies done previously.^[9,15]^ Other reported causative agents include Diphtheroids (e.g., *Corynebacterium* species and *Propionibacterium acnes*), and Gram-negative bacilli, which were also encountered in our study (e.g., *Pseudomonas aeruginosa*). *C albicans* has also been reported to be a causative agent in a minority of cases, and other more rare etiologies include other types of fungi and nontuberculous mycobacterium.^[16]^ We also encountered one culture-negative CIED infection, which has previously been reported in 7% to 16% of cases.^[6,17]^*Achromobacter xylosoxidans* was seen in one of the polymicrobial infections and has been previously reported to be associated with CIED infection.^[18]^

**Figure 3:**
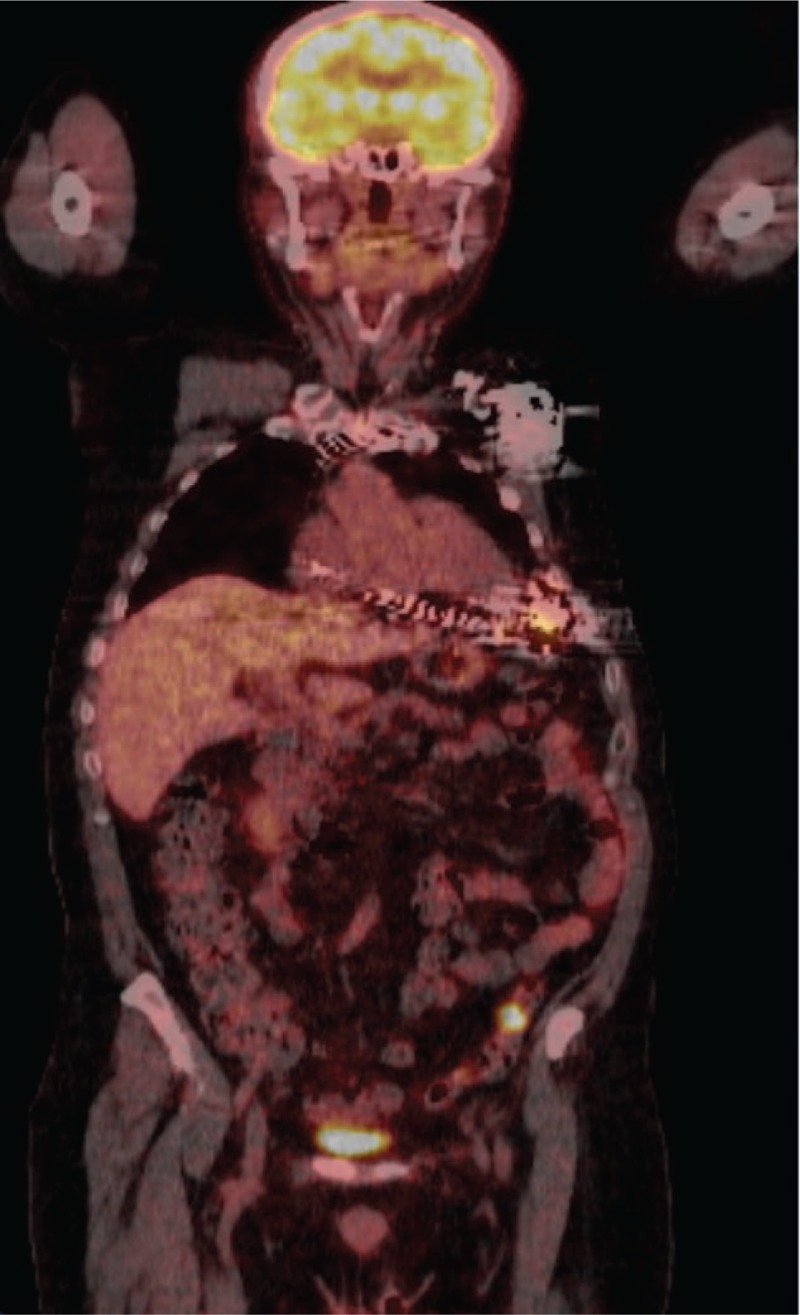
Positron emission tomography-computed tomography scan of patient presenting with *Sphingomonas paucimobilis* cardiac implantable electronic device infection.

The unusual and rare pathogens causing CIED infection encountered in this study include *B melitensis*, *S paucimobilis*, and *K schroeteri.* Brucella is endemic in Lebanon. It can present acutely, subacutely, or chronically with various clinical symptoms. The most commonly encountered symptoms include fever, sweating, fatigue, and diffuse aches. Neurologic and articular complications are often seen as well. The disease is predominant in developing countries and diagnosis is challenging because it can present in any organ and its symptoms can overlap with a variety of infectious and noninfectious disorders.^[19]^ Brucella*-*specific tests are therefore important in endemic regions. Endemic organisms to different regions of the world must be considered whenever there is diagnostic challenge in the microbial etiology of infection. The second unusual etiology of CIED infection encountered in this study is *S paucimobilis*, a Gram-negative bacillus that is a widespread cause of nosocomial opportunistic infections, including bacteremia.^[20]^ It is seen in wood chips of coniferous trees.^[21]^ One of the patients with *S paucimobilis* infection is a carpenter, and this stresses the importance of occupational exposure in detecting the etiology of infection. Positron emission tomography-computed tomography scan aided in the diagnosis of CIED infection in this patient and is shown in Figure 3. Combined PET and CT is useful in detecting and localizing infection, and can help differentiate between soft tissue infection overlying the generator and generator infection.^[4]^*K schroeteri* is a relatively novel species introduced by Becker et al in 2002.^[22]^ Little is known about its natural presence, but it has been in rare cases been found in the blood and prosthetic heart valves.^[23,24]^ The emerging case reports on this organism since its discovery may indicate its affinity to foreign bodies.

Diabetes and CKD were among the most common comorbidities observed, however, only one patient was immunosuppressed, due to CLL. In a study by Ipek et al, immune suppression was a prominent comorbidities associated with CIED infection, along with diabetes, heart failure, and hypertension. Anemia was also a comorbidity involved with CIED infection.^[25]^ Smoking and anticoagulation therapy were also commonly seen in our cohort of patients. Anticoagulation may increase risk of infection due to hematoma formation and poor wound healing.^[1]^ CKD is one of the strongest risk factors for CIED infection, especially since it is associated with immune dysfunction.^[26]^ Our data demonstrates CKD in half of the cohort of patients, further strengthening the association of CKD and CIED infection. Infections were also more commonly seen in patients with advanced age (>60). The majority of patients had normal white blood cell counts on presentation making its diagnostic usefulness uncertain.

CIED infection often poses a diagnostic challenge to physicians due to the wide variety of presentations. CIED pocket infection can be easily suspected in cases where local inflammatory signs are present, although it could be challenging in the early postoperative period where hematoma formation can mimic a pocket infection.^[6]^ This is not the case when local signs of infection are absent or minimal, therefore, a high degree of clinical suspicion is required, especially when patients present with vague symptoms such as malaise, fatigue, anorexia, or fever. The most common presentation of CIED infection is generator pocket infection, which was seen in 59.1% of patients in this sample. A retrospective review of 189 patients with CIED infection done by Sohail et al showed that 69% of patients presented with generator pocket infection.^[27]^ Suggestive physical findings include erythema, edema, tenderness, discharge, or ulceration at the site of infection.^[1]^ Skin changes were noted in 86.4% of patients. Advanced skin presentations suggest that earlier signs of infection might have been initially missed. This highlights the importance of continuous monitoring of patients’ wounds even long after CIED implantation, and need for educating patients to observe closely their wounds on a daily basis for any signs of skin changes or discharge. In some cases, where no skin changes are present, CIED infection becomes more of a diagnostic challenge. CIED infection can present as infective endocarditis which involves the endovascular portion of the device. In these cases, systemic signs including fever, anorexia, and malaise are usually present.^[1]^ Diagnosis would require the presence of lead or valvular vegetations on echocardiography or meeting the modified Duke criteria for infective endocarditis.^[11]^ Infective endocarditis was seen in 13.6% of our patients and for comparison, 23% of patients presented similarly in a study conducted by Lekkerkerker et al.^[8]^ In some cases, as seen in one of our patients with culture-negative CIED infection site, positive blood cultures may be the sole manifestation of CIED infection without any evidence of pocket infection or vegetations on echocardiographic evaluation.^[1]^ Time of onset of device infection has been variable between different studies; 9.1%, 13.6%, and 77.3% of our patients had CIED infection 0 to 28 days, between 28 and 356 days, and after 356 days of implantation, respectively. Chua et al reviewed 129 patients with CIED infection and found that 25%, 33%, and 42% of patients developed infection 0 to 28 days, between 28 and 356 days, and after 356 days of implantation, respectively.^[28]^ In a study conducted on 24 patients with CIED infection, 60% were diagnosed within 90 days of their most recent intervention.^[29]^

Antibiotics are given prophylactically for CIED implantation to prevent future infection. All patients had antibiotic prophylaxis prior to device implantation, reinforcing the fact that this preventive measure is not enough in completely preventing future CIED infections. In general, management of CIED infection involves three key steps. First, the diagnosis must be confirmed. Second, all hardware must be removed, and third, systemic antimicrobials should be administered. Selection of appropriate antimicrobial therapy should be based on the pathogen identified and the results of in vitro susceptibility testing. Since the majority of infections are due to staphylococcus species, empirical therapy with vancomycin is considered appropriate, and once the organism is identified, targeted therapy is warranted.^[1]^ If needed, reimplantation of a new device may be done through an uninfected route. The majority of our patients were started on vancomycin therapy and all of them had their devices removed and were appropriately treated with antimicrobial therapy until the infection resolved. In general, from a procedural standpoint, vancomycin would be administered 1 h prior to procedure, and during the procedure, the created pocket would be irrigated with gentamicin. Eight patients required reimplantation of their devices. Review of the literature suggests that up to half of patients will not require reimplantation of their devices.^[26]^

**Figure 4:**
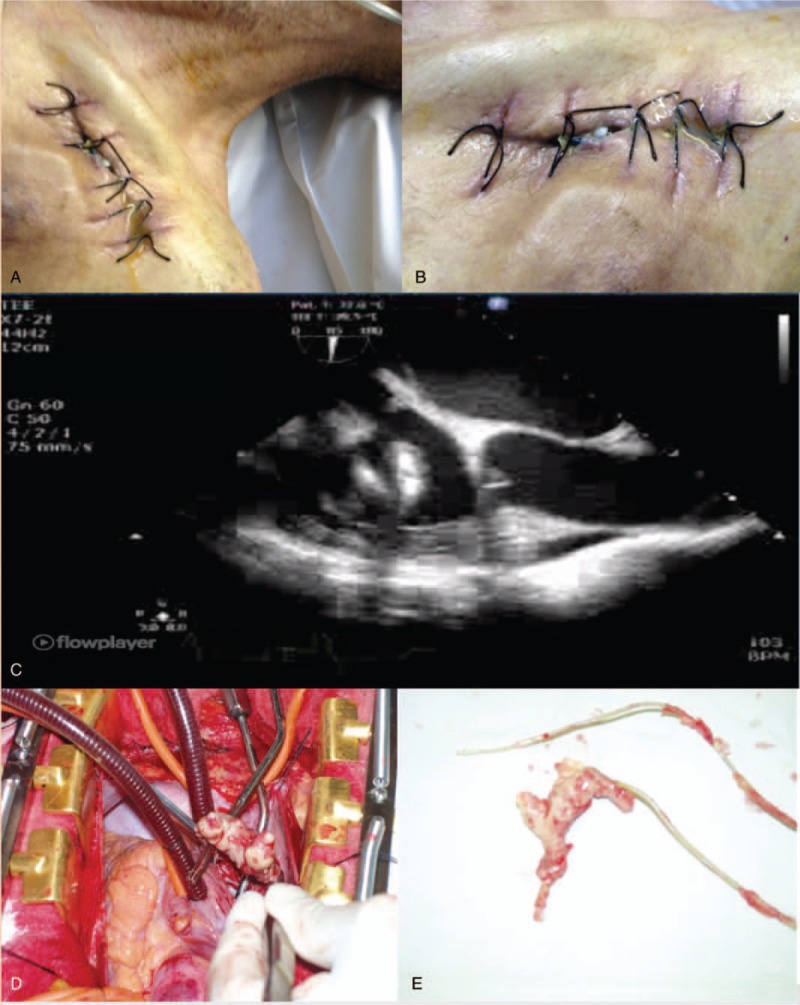
Skin changes seen in patient presenting with *Enterobacter cloacae* cardiac implantable electronic device infection (Panels A and B), septic shock and a large vegetation on transesophageal echocardiography (Panel C) on the lead that was surgically removed (Panels D and E).

Outcomes depend mainly on the type of infection and on patient comorbidities. In-hospital mortality rate associated with pocket infections is less than 5%, but it may be as high as 29% if not properly treated and if bacteremia or endovascular involvement is present.^[30]^ Overall mortality after having CIED extracted has been reported to be between 2.6% and 18%.^[31]^ In our study, one patient passed away following CIED extraction, but more long-term follow-up is warranted. The patient who passed away had a Gram-negative bacillus (*E cloacae*) CIED infection, septic shock, and a large vegetation on the lead that was surgically removed (Fig. 4). Increased mortality in the setting of CIED infection was noted by Athan et al, when an associated infective endocarditis is found.^[32]^

The advances in noninvasive modeling in the heart and pre-procedural imaging can help the electrophysiologist in the lead extraction procedure necessary after lead infection. These noninvasive modeling techniques have also helped in the diagnosis and treatment of arrhythmia. Wang et al used volumetric myocardial transmembrane potential dynamics to exhibit details of arrhythmogenic substrates in the myocardium.^[33]^

The major limitation to this study is the relatively small sample size, which makes it difficult to determine significant association between variables. Another limitation is the retrospective nature of the study as data was solely obtained from medical charts. Finally, this study is only a single-center study. A multicenter study could be more appropriate to obtain a better population sample and therefore make the study more broadly applicable.

## 5 Conclusion

This is the first study to bring forth CIED infection data from Lebanon, and while it is only a single-centered study, it is a stepping-stone in the close monitoring of CIED infections in Lebanon in an attempt to prevent the serious complication of CIED infection. Unique cases must be reported for other physicians to be made aware of potential microbial etiologies of infection and therefore help in implementing therapeutic strategies. Regional registries of etiologies of CIED infection may help in facilitating the diagnostic process.

Prevention of CIED infection is important in the management of patients selected for device implantation. Therefore, prior to CIED placement, the physician must look for any clinical signs of infection in order to determine the appropriate timing of the procedure. Administration of intravenous antibiotic 1 h before implantation is recommended according to several studies. Most experts recommend the use of a first generation cephalosporin such as cefazolin. Although not generally recommended, some experts advocate the use of vancomycin instead, and it is usually given 90 to 120 min before the procedure. While antibiotic prophylaxis is important, it does not entirely prevent infection. Other measures to prevent CIED infection include: preoperative antiseptic preparation of the skin, intraprocedural sterile techniques, sterile covering of the image intensifier and prevention of hematoma formation. Worldwide trials are being conducted on antibiotic envelopes implanted around the devices to potentially help reduce CIED infection.^[34]^ Early follow-up, mainly in patients with multiple comorbidities, and thorough patient education are of great importance for early identification of device infection.

## Acknowledgments

None.

## Author contributions

**Conceptualization:** Marwan Refaat, Patrick Zakka, Maurice Khoury.

**Data curation:** Patrick Zakka, Maurice Khoury, Hassan Chami, Shareef Mansour, Bernard Harbieh, Bernard Abi-Saleh.

**Formal analysis:** Patrick Zakka.

**Investigation:** Marwan Refaat, Patrick Zakka, Maurice Khoury, Hassan Chami.

**Methodology:** Patrick Zakka, Maurice Khoury.

**Project administration:** Marwan Refaat, Patrick Zakka.

**Resources:** Marwan Refaat, Patrick Zakka, Shareef Mansour.

**Supervision:** Marwan Refaat, Maurice Khoury, Bernard Abi-Saleh, Abdul Rahman Bizri.

**Writing – original draft:** Patrick Zakka, Hassan Chami, Shareef Mansour.

**Writing – review & editing:** Marwan Refaat, Patrick Zakka, Maurice Khoury, Bernard Harbieh, Bernard Abi-Saleh, Abdul Rahman Bizri.

Patrick Zakka: 0000-0002-1762-3517

Patrick Zakka orcid: 0000-0002-1762-3517.
